# Serum level of monocyte chemotactic protein 1, N-terminal brain natural peptide in patients with coronary heart disease after nutritional changes

**DOI:** 10.5937/jomb0-55845

**Published:** 2025-10-28

**Authors:** Yueyou Ding, Wenhui Ji, Hongchao Zheng, Zhancheng Wang

**Affiliations:** 1 Shanghai Eighth People's Hospital, Department of Cardiology, Shanghai, 200235, China; 2 Huajing Community Health Service Centre for Xuhui District, Department of Internal Medicine, Shanghai, 200231, China; 3 Central Hospital of Xuhui District, Department of Cardiology, Shanghai, 200231, China

**Keywords:** NT-proBNP, MCP-1, Baoyuan decoction, Taohong Siwu decoction, Western medicine, elderly coronary heart disease, LDL-C, TG, TC, HDL-C, NT-proBNP, MCP-1, Baoyuan dekokt, Taohong Siwu dekokt, zapadna medicina, starija populacija, koronarna bolest srca, LDL-C, TG, TC, HDL-C

## Abstract

**Background:**

Coronary heart disease (CHD) is a leading cause of morbidity among elderly populations, with inflammation and cardiac dysfunction indicated by elevated MCP-1 and NT-proBNP levels. This study evaluated the effects of integrating Traditional Chinese Medicine (Baoyuan and Taohong Siwu decoctions) with standard Western therapy on serum MCP-1 and NT-proBNP in elderly CHD patients. Results demonstrated significant reductions in these biomarkers, supporting the complementary role of TCM in managing CHD.

**Methods:**

A total of 90 elderly CHD patients were randomly allocated into two groups (n=45 each): the control group (CG), receiving conventional Western medicine alone, and the research group (RG), treated with BYD-THSWD combined with standard Western pharmacotherapy. Serum levels of MCP-1 and NT-proBNP, lipid profiles (TG, TC, LDL-C, HDL-C), and clinical symptoms (chest pain, chest tightness, fatigue, sweating) were assessed at baseline and after 1, 2, and 3 months of treatment. Statistical comparisons between groups were conducted using independent-sample t-tests and chi-square tests.

**Results:**

After 3 months, serum levels of MCP-1 (113.09±5.49 vs. 126.38±7.04 pg/mL, P&lt;0.05) and NT-proBNP (614.28±54.77 vs. 781.28±68.29 ng/mL, P&lt;0.05) were significantly lower in the RG compared to the CG. Similarly, the RG exhibited significantly improved lipid profiles and greater symptomatic relief, reflected by significantly lower TCM symptom scores for chest pain, chest tightness, fatigue, and sweating compared to the CG at all post-treatment intervals (all P&lt;0.05).

**Conclusions:**

Integrating Baoyuan decoction and Taohong Siwu decoction with conventional Western medicine significantly reduces MCP-1 and NT-proBNP levels, improves lipid metabolism, and alleviates clinical symptoms in elderly coronary heart disease patients. These findings highlight the potential of Traditional Chinese Medicine as a complementary therapy in enhancing standard CHD treatment outcomes.

## Abstract (sr)

**Uvod:**

Koronarna bolest srca (KBS) je jedan od vodećih uzroka morbiditeta među starijom populacijom, pri čemu su upala i srčana disfunkcija povezane sa povišenim nivoima MCP-1 i NT-proBNP. Ova studija je procenila efekte integracije tradicionalne kineske medicine (Baoyuan i Taohong Siwu dekokti) sa standardnom zapadnom terapijom na serumske nivoe MCP-1 i NT-proBNP kod starijih pacijenata sa KBS. Rezultati su pokazali značajno smanjen-je ovih biomarkera, što podržava komplementarnu ulogu tradicionalne kineske medicine u lečenju KBS-a.

**Metode:**

U studiju je bilo uključeno ukupno 90 starijih paci-jenata sa KBS-om, koji su nasumično raspoređeni u dve grupe (n=45): kontrolnu grupu (CG), koja je primala isključivo konvencionalnu zapadnu terapiju, i grupu ispi-tanika (RG), koja je uz standardnu farmakoterapiju dobijala i BYD-THSWD. Serumski nivoi MCP-1 i NT-proBNP, lipidni profil (TG, TC, LDL-C, HDL-C) i klinički simptomi (bol u grudima, stezanje u grudima, umor, znojenje) procenjivani su na početku studije i nakon 1, 2 i 3 meseca lečenja. Statističke analize između grupa sprovedene su pomoću t-testa za nezavisne uzorke i hi-kvadrat testa.

**Rezultati:**

Nakon 3 meseca, serumski nivoi MCP-1 (113,09±5,49 vs. 126,38±7,04 pg/mL, P&lt;0,05) i NT-proBNP (614,28±54,77 vs. 781,28±68,29 ng/mL, P&lt;0,05) su bili su značajno niži u RG u poređenju sa CG. Takođe, RG je pokazala značajno poboljšane vrednosti lipidnog profila i veće ublažavanje simptoma, što se ogledalo u nižim TCM simptomatskim skorovima za bol u grudima, stezanje u grudima, umor i znojenje u svim peri-odima nakon terapije (svi P&lt;0,05).

**Zaključak:**

Integracija Baoyuan dekokta i Taohong Siwu dekokta sa konvencionalnom zapadnom terapijom značajno smanjuje nivoe MCP-1 i NT-proBNP, poboljšava metabolizam lipida i ublažava kliničke simptome kod stari-jih pacijenata sa koronarnom bolešću srca. Ovi nalazi ističu potencijal tradicionalne kineske medicine kao komplemen-tarne terapije u unapređenju ishoda standardnog lečenja KBS-a.

## Introduction

Coronary heart disease (CHD) remains a leading cause of morbidity and mortality globally, particularly among elderly populations [1] [2] [3]. It is characterised by atherosclerotic narrowing of coronary arteries, causing myocardial ischemia and subsequent deterioration of cardiac function [4]. Aging populations often exhibit additional risk factors such as hyperlipidemia, hypertension, and diabetes mellitus, further complicating disease management [5]. Effective treatment strategies are essential to mitigate disease progression, improve cardiac function, and enhance the quality of life in elderly CHD patients [3].

Recent research has emphasised inflammation as a key pathogenic mechanism in the development and progression of CHD. Monocyte chemotactic protein-1 (MCP-1), a member of the C-C chemokine family, has attracted attention due to its pivotal role in recruiting monocytes to sites of inflammation, contributing to vascular damage and plaque formation [6]. Elevated MCP-1 levels have consistently been associated with increased risks of atherosclerosis, plaque vulnerability, and acute coronary events [7]. Additionally, MCP-1 is considered an early biomarker for predicting adverse cardiovascular outcomes, reflecting the severity of inflammation within coronary lesions [8].

Similarly, N-terminal pro-brain natriuretic peptide (NT-proBNP) is a widely recognised biomarkerreflecting cardiac dysfunction, particularly ventricular strain and volume overload [9]. NT-proBNP is secreted primarily from ventricular cardiomyocytes in response to myocardial stress and stretching, playing a crucial role in cardiovascular homeostasis by regulating natriuresis, diuresis, vasodilation, and inhibition of the renin-angiotensin-aldosterone system [10]. Previous studies have consistently demonstrated that elevated serum NT-proBNP levels are strong predictors of mortality, heart failure, and overall adverse cardiac events in CHD patients [11] [12]. Thus, measuring serum NT-proBNP levels is essential for assessing the cardiac function status and prognosis of patients with coronary heart disease.

Current therapeutic strategies for elderly CHD patients primarily involve pharmacological interventions such as antiplatelet agents, lipid-lowering drugs, and vasodilators, which aim to prevent thrombosis, manage dyslipidemia, and improve coronary circulation [13] [14]. Despite the efficacy of Western medicines, a significant number of elderly patients continue to experience recurrent angina, poor lipid control, and persistent inflammatory states, highlighting the need for integrated therapeutic approaches.

Traditional Chinese Medicine (TCM), mainly herbal decoctions, is increasingly employed as anadjunct therapy in cardiovascular disease treatment due to its multi-targeted therapeutic effects, minimal side effects, and potential for improving patients’ overall condition [15]. Among numerous TCM formulas, Baoyuan decoction (BYD) and Taohong Siwu decoction (THSWD) are particularly prominent. BYD primarily functions through invigorating qi, strengthening myocardial energy metabolism, and improving cardiac function. At the same time, THSWD is noted for its effects on invigorating blood circulation, eliminating blood stasis, reducing inflammation, and protecting myocardial cells [16] [17]. Clinical studies have indicated that these decoctions effectively improve symptoms such as chest tightness, fatigue, chest pain, and shortness of breath, significantly enhancing patient outcomes.

However, despite growing evidence supporting the clinical benefits of TCM in CHD management,there remains limited information on the specific biochemical changes induced by these nutritional and medicinal therapies. Particularly, the impact of BYDTHSWD combined with standard Western medicine on critical cardiovascular biomarkers, such as MCP-1 and NT-proBNP, has not yet been thoroughly explored or clearly defined.

The purpose of this study, therefore, was to examine the effect of nutritional modifications incorporating Baoyuan decoction and Taohong Siwu decoction in combination with standard Western pharmacotherapy on serum levels of MCP-1 and NT-proBNP in elderly patients diagnosed with coronary heart disease. Understanding how these traditional therapies influence inflammatory and cardiac biomarkers could provide insights into their potential mechanisms of action and inform clinical decisions regarding integrated treatments for improved cardiovascular outcomes in elderly CHD patients.

## Materials and methods

### Research subjects

This study involved 90 elderly patients diagnosed with coronary heart disease (CHD) whoreceived medical treatment at our institution from January 2023 to December 2024. Patients wereevenly divided into two treatment groups (45 patients per group). Demographic characteristics and clinical conditions – including gender distribution, average age, body mass index (BMI), height, duration and severity of CHD, presence of comorbid conditions, and CHD grading – were statistically comparable between the two groups (P>0.05), as detailed in Table 1.

**Table 1:** Comparison of baseline data (x̄±s, %).

Group	n	Sex		Age<br>(year old)	BMI<br>(kg/m^2^)	Height (m)	Disease course<br>(year)
		Male	Female				
CG	45	26	19	62.49±1.27	23.54±2.06	1.67±0.15	3.56±1.16
RG	45	23	22	62.71±1.36	23.91±1.86	1.70±0.11	3.81±1.02
*t*		0.403		0.793	0.894	1.082	1.086
*P*		0.525		0.430	0.374	0.282	0.281
Group	n	Merged underlying diseases			CHD grade		
		Hypertension	Hyperlipidemia	Diabetes	Level	Level	Level
CG	45	12	15	18	27	13	5
RG	45	14	11	20	23	15	7
*t*		0.875			0.796		
*P*		0.646			0.672		

### Inclusion criteria

(A) Patients diagnosed with coronary heart disease meet established diagnostic standards as defined by current clinical guidelines (reference 7).

(B) Patients receiving pharmacological treatment for CHD for the first time at our medical centre.

(C) Traditional Chinese Medicine (TCM) diagnostic standards confirm that the patient’s condition is consistent with Qi deficiency and blood stasis syndrome.

(D) Clinical documentation indicating the initial administration of CHD medications that occurred at our institution is available.

(E) Patients exhibiting stable cardiac function classified at levels I–III according to NYHA classification guidelines.

### Exclusion criteria

(A) History of acute myocardial infarction or unstable angina episodes within the last 6 months.

(B) Cardiac functional classification of IV or higher (indicating severe cardiac insufficiency).

(C) Documented chronic abuse of drugs or alcohol.

(D) Severe comorbid diabetes mellitus and hypertension that cannot be effectively managed or stabilised.

(E) Co-existing severe hemorrhagic conditions, severe cerebrovascular disease, or active malignancies.

(F) History of severe psychiatric disorders or other conditions affecting adherence and cooperation in medical treatment protocols.

### Treatment protocols

Control Group (CG): Patients received standardised Western medical therapy, including:

Betaloc (Metoprolol): Oral dose of 25–50 mg administered twice daily.

Aspirin and lipid-lowering therapy: Patients received Betaloc at doses of 25–50 mg twice daily orally, Rosuvastatin 10–20 mg administered orally once nightly, and additional medications as clinically indicated.

Each therapeutic course lasted one month, with a total treatment duration of three consecutive months.

Research Group (RG): Patients in the RG received standard Western medications identical tothe Control Group, supplemented with the herbal formula BYD-THSWD tailored to Qi deficiency and blood stasis syndrome:

The BYD-THSWD prescription comprised:

Ophiopogon japonicus (20 g), Ligusticum wallichii (9 g), Astragalus membranaceus (20 g), Caulis spatholobi (15 g), Angelica sinensis (10 g), Radix liquiritiae (6 g), Safflower (10 g), Fried semen ziziphi spinosae (15 g), Semen ziziphi spinosae (fried, 15 g), Angelica sinensis (10 g), Ligusticum wallichii (9 g), Ophiopogon japonicus (20 g), and Safflower (10 g).

Additional herbal ingredients were customised based on presenting symptoms:

For patients experiencing pronounced fatigue, 15 g yam and 15 g fried Atractylodes macrocephala Koidz were included.

In cases of severe insomnia, 15 g tube of flueflower stem was added.

For significant chest tightness, 15 g Fructus Aurantii was supplemented.

The herbs were prepared through water decoction, with patients consuming one dose daily.

### Evaluation indices and procedures

Clinical assessment criteria included:

Symptom severity scoring (0–3 scale): Patients were assessed for symptom severity in terms of clinical manifestations such as chest pain, shortness of breath, fatigue, and insomnia, as rated on a four-point scale ranging from 0 (no symptoms) to 3 (severe symptoms). Higher cumulative scores indicated more severe clinical presentations.

Blood lipid profiling: Fasting venous blood samples (3 mL) were collected in the morning to measure triglycerides (TG), total cholesterol (TC), low-density lipoprotein cholesterol (LDL-C), and high-density lipoprotein cholesterol (HDL-C). Samples were analysed promptly post-collection.

Serum biomarkers of cardiac function and inflammation: Venous blood (5 mL) was drawn after overnight fasting to determine the levels of NT-proBNP and MCP-1. NT-proBNP was measured via electrochemiluminescence immunoassay (ECLIA), while MCP-1 levels were evaluated using enzyme-linked immunosorbent assay (ELISA) [18] [19].

### Statistical analysis

Statistical analyses were conducted using SPSS software (version 26.0). Continuous data were expressed as mean ± standard deviation (x̄ ± s), while categorical data were presented as frequencies and percentages. Differences between groups were assessed using independent-sample t-tests for normally distributed continuous data. Chi-square tests were utilised to evaluate categorical variables. A p-value less than 0.05 (P<0.05) was defined as statistically significant, indicating meaningful differences between the two groups.

## Results

The study involved 90 elderly patients diagnosed with coronary heart disease (CHD), with a mean age of approximately 62 years. Participants were randomly assigned to two groups: the control group (CG) receiving conventional Western medicine and the research group (RG) receiving a combination of Baoyuan and Taohong Siwu decoctions along with Western therapy. The baseline characteristics, including gender, body mass index (BMI), height, disease duration, and severity of CHD, were comparable between the two groups. Both groups included individuals with comorbid conditions such as hypertension, hyperlipidemia, and diabetes, with no significant differences in these conditions or the severity of CHD across groups. The study was conducted over a period from January 2023 to December 2024, and all participants provided informed consent to participate.

### Comparison of baseline data

The demographic and clinical baseline characteristics of participants in both the control group (CG)and research group (RG) were comparable. There were no statistically significant differences between the two groups concerning gender distribution, age, BMI, height, duration of illness, prevalence of underlying diseases (hypertension, hyperlipidemia, diabetes), or coronary heart disease (CHD) severity grading (all P>0.05).

### TCM symptom scores

Both groups had similar Traditional Chinese Medicine (TCM) symptom scores prior to treatment (P>0.05). However, after treatment at different time points, symptoms, including chest pain, chest tightness, fatigue, and sweating, significantly improved in both groups. Notably, the improvements were more significant in the RG compared with the CG at each time interval (1 month, 2 months, and 3 months after treatment), with statistically significant differences (P<0.05). Table 2 Table 3

**Table 2:** TCM Symptom Scores for Two Groups (x̄±s, point). Note: * Compared with before treatment, P<0.05.^ *#^ Compared with the 1-month treatment, P<0.05. ^*#&^ Compared with the 2-month treatment, P<0.05.

Group	n	Chest pain				Chest tightness			
		Before	1 month after	2 months	3 months after	Before	1 month	2 months	3 months
CG	45	2.48±0.48	2.01±0.34^*^	1.87±0.30^*^	1.69±0.19^*#&^	2.51±0.38	1.99±0.25^*^	1.81±0.17^*^	1.65±0.14^*^
RG	45	2.45±0.51	1.85±0.29^*^	1.68±0.22^*^	1.31±0.12^*#&^	2.61±0.27	1.80±0.21^*^	1.73±0.15^*^	1.43±0.10^*^
*t*		0.287	2.402	3.426	11.343	1.439	3.904	2.367	8.578
*P*		0.775	0.018	0.001	0.000	0.154	0.002	0.020	0.000
	n	Feeble				Sweating			
		Before	1 month	2 months	3 months after	Before	1 month	2 months	3 months after
CG	45	2.71±0.26	1.98±0.23^*^	1.76±0.15^*^	1.51±0.11^*#&^	2.44±0.71	1.93±0.54^*^	1.78±0.43^*^	1.34±0.34^*^
RG	45	2.45±0.29	1.80±0.16^*^	1.55±0.12^*^	1.30±0.03^*#&^	2.38±0.65	1.71±0.41^*^	1.41±0.29^*^	1.02±0.19^*^
*t*		4.478	4.310	7.334	12.355	0.418	2.177	4.786	5.511
*P*		0.000	0.000	0.000	0.000	0.677	0.032	0.000	0.000

**Table 3:** TG, TC, LDL-C, and HDL-C (x̄±s, mmol/L). Note: * Compared with before treatment, P<0.05. ^*#^ Compared with the 1-month treatment, P<0.05. ^*#&^ Compared with the 2-month treatment, P<0.05.

Group	n	TG				TC			
		Before	1 month<br>after	2 months<br>after	3 months<br>after	Before	1 month<br>after	2 months<br>after	3 months<br>after
CG	45	6.82±2.13	5.42±1.78^*^	4.00±1.39^*#^	2.97±1.10^*#&^	6.49±2.08	5.11±1.85^*^	4.13±1.57^*#^	3.10±0.72^*#&^
RG	45	6.50±2.20	4.06±1.56^*^	3.06±1.18^*#^	1.26±0.67^*#&^	6.63±2.17	4.20±1.69^*^	3.24±1.31^*#^	2.19±0.39^*#&^
*t*		0.701	3.855	3.458	8.906	0.312	2.436	2.920	7.455
*P*		0.485	0.000	0.001	0.000	0.755	0.017	0.004	0.000
	n	LDL-C				HDL-C			
		Before	1 month<br>after	2 months<br>after	3 months<br>after	Before	1 month<br>after	2 months<br>after	3 months<br>after
CG	45	7.63±1.99	6.00±1.57^*^	5.97±1.45^*#^	4.18±1.00^*#&^	6.15±1.01	5.16±0.87^*^	4.76±0.51^*#^	3.00±0.26^*#&^
RG	45	7.71±1.78	5.01±1.31^*^	4.59±1.13^*#^	3.10±0.51^*#&^	6.26±1.07	4.79±0.75^*^	3.53±0.31^*#^	1.23±0.04^*#&^
*t*		0.201	3.248	5.036	6.454	0.501	2.161	13.825	45.136
*P*		0.841	0.002	0.000	0.000	0.617	0.033	0.000	0.000

### Comparison of TG, TC, LDL-C, and HDL-C

Initially, no significant differences existed between RG and CG for triglycerides (TG), total cholesterol (TC), low-density lipoprotein cholesterol (LDL-C), or high-density lipoprotein cholesterol (HDL-C) (P>0.05). Post-treatment, both groups showed significant reductions in these lipid parameters. Notably, after 3 months of treatment, RG (treated with BYD-THSWD combined with Western medicine) exhibited significantly lower levels of TG, TC, LDL-C, and HDL-C compared to CG (treated with Western medicine alone) (all P<0.05).

**Figure 1:**
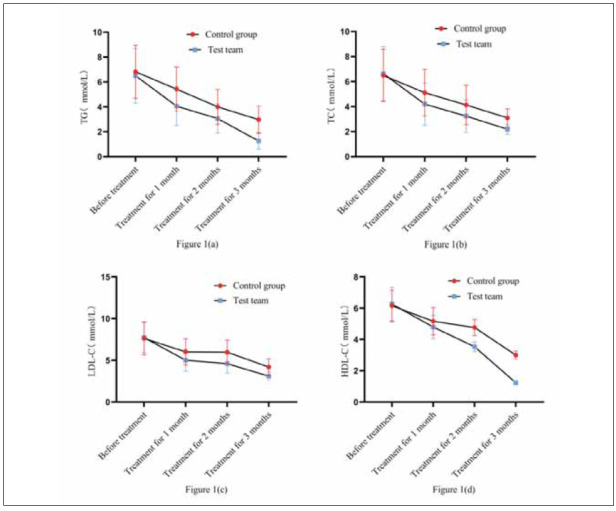
Comparison of TG, TC, LDL-C, and HDL-C.<br>Note: Figure 1(a) shows TG, Figure 1(b) shows TC, Figure 1(c) shows LDL-C, and Figure 1(d) shows HDL-C. The red circular curverepresents CG. The blue square curve represents RG. The curve fluctuation indicates the changes in various blood lipid indicators before and after treatment in both groups.

Compared with CG treated with Western medicine alone, RG with BYD-THSWD showed a decreasein TG, TC, LDL-C, and HDL-C levels. Figure 1(a), Figure 1(b), Figure 1(c), and Figure 1(d) showed the trend changes of various indicators. Two groups had the same TG, TC, LDL-C, and HDL-C levels before treatment (*P*>0.05) and were treated for 1 and 2 months. After 3 months, the levels of TG, TC, LDL-C, and HDL-C in RG were lower than CG’s (*P*<0.05). Table 4

**Table 4:** Comparison of NT-proBNP and MCP-1 (x̄±s). Note: * Compared with before treatment, P<0.05. *# Compared with the 1-month treatment, P<0.05. *#& Compared with the 2-month treatment, P<0.05.

Group	n	MCP-1 (pg/mL)				NT-proBNP (ng/mL)			
		Before	1 month<br>after	2 months<br>after	3 months<br>after	Before	1 month<br>after	2 months<br>after	3 months<br>after
CG	45	156.34±<br>12.74	141.06±<br>11.06^*^	132.18±<br>8.37^*#^	126.38±<br>7.04^*#&^	1024.52±<br>123.09	916.34±<br>110.34^*^	816.39±<br>84.37^*#^	781.28±<br>68.29^*#&^
RG	45	156.19±<br>12.37	135.29±<br>10.09^*^	127.34±<br>7.16^*#^	113.09±<br>5.49^*#&^	1024.37±<br>123.13	804.28±<br>98.74^*^	700.25±<br>71.04^*#^	614.28±<br>54.77^*#&^
*t*		0.057	2.585	2.948	3.000	0.006	5.077	7.064	12.797
*P*		0.955	0.011	0.004	0.004	0.995	0.000	0.000	0.000

### Comparison of Serum Factors NT-proBNP and MCP-1

No significant differences in cardiac biomarkers (NT-proBNP and MCP-1) were observed between groups at baseline (P>0.05). After treatment durations of 1, 2, and 3 months, both groups displayed reductions in NT-proBNP and MCP-1. However, the RG experienced more pronounced decreases compared to the CG at all post-treatment time points, demonstrating statistically significant differences (all P<0.05). This indicates that combination therapy (BYD-THSWD plus Western medicine) provided greater benefits in reducing serum NT-proBNP and MCP-1 than Western medicine alone.

**Figure 2:**
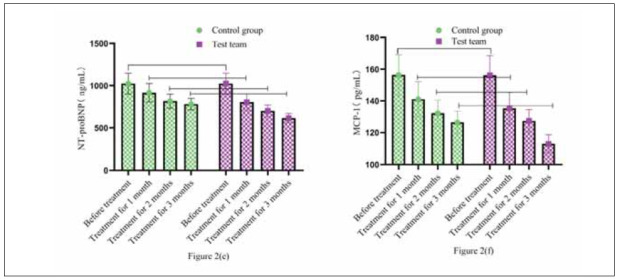
Comparison of serum factors NT-proBNP and MCP-1.<br>Note: Figure 2(e) shows NT-proBNP, and Figure 2(f) shows MCP-1. The green circular bar chart is CG. The purple square bar chart shows RG. The high and low levels of the bar chart represent the changes in various serum indicators before and after treatment in both groups.

Compared to the control group (CG), which received only Western medicine, the research group (RG) treated with BYD-THSWD combined with Western medicine exhibited more significant reductions in serum NT-proBNP and MCP-1 levels. Figure 2(e) and Figure 2(f) illustrate the trends of these indicators throughout treatment. Initially, no significant differences were found between the two groups (P>0.05). However, after treatment at various time points, NT-proBNP and MCP-1 were significantly lower in RG than in CG (P<0.05) (Figure 2).

## Discussion

This study demonstrated that elderly patients with coronary heart disease (CHD) treated with acombination of Baoyuan decoction (BYD), Taohong Siwu decoction (THSWD), and standard Western medical therapy experienced significant improvements in both clinical symptoms and biochemical markers compared to those receiving Western medication alone. The primary findings included a marked reduction in serum levels of monocyte chemotactic protein-1 (MCP-1) and N-terminal probrain natriuretic peptide (NT-proBNP), along with substantial improvements in lipid profiles (TG, TC, LDL-C, HDL-C). Additionally, significant symptomatic relief, including decreased chest pain, chest tightness, fatigue, and sweating, was observed in the integrated therapy group. These results demonstrate that incorporating BYD-THSWD into conventional Western medicine can effectively enhance cardiac function, alleviate inflammation, and improve lipid metabolism. This suggests a beneficial adjunctive role for Traditional Chinese Medicine in managing elderly CHD patients.

The observed significant reduction in MCP-1 levels among patients receiving combined treatment aligns with the growing body of evidence highlighting the critical role of inflammation in CHD pathogenesis. Elevated MCP-1 is well-established as an early inflammatory marker predictive of increased cardiovascular risk, reflecting the active inflammatory status and plaque vulnerability within coronary arteries. By substantially decreasing MCP-1, BYD-THSWD appears to exert anti-inflammatory effects, potentially reducing atherosclerotic progression and enhancing plaque stability. The mechanism underlying this benefit may involve herbal components known for their anti-inflammatory and antioxidative properties, particularly Astragalus membranaceus and Angelica sinensis, which are integral constituents of the BYD-THSWD formula. Thus, the improvement in MCP-1 provides biochemical evidence supporting the clinical efficacy of BYD-THSWD in managing inflammatory responses associated with coronary heart disease.

In a study by Martín-Reyes et al. [20], elevated plasma levels of monocyte chemoattractant protein-1 (MCP-1) and N-terminal pro-brain natriuretic peptide (NT-proBNP) were independently associated with greater complexity of coronary artery disease (CAD), as measured by the Syntax Score. Additionally, low calcidiol (vitamin D metabolite) levels predicted more severe coronary artery calcification, indicating its role in mineral metabolism and CAD progression. Our findings align with these results, as we similarly observed elevated MCP-1 and NT-proBNP levels among elderly coronary heart disease (CHD) patients. Importantly, our study demonstrated that combining Traditional Chinese Medicine (Baoyuan and Taohong Siwu decoctions) with conventional Western treatment significantly reduced MCP-1 and NT-proBNP levels, improved lipid profiles, and alleviated clinical symptoms. While Martín-Reyes et al. highlighted biomarker associations, our findings suggest that integrative therapeutic approaches could actively reduce these markers and potentially impact CAD severity. Future studies should investigate the influence of TCM on biomarkers like calcidiol further to understand its therapeutic benefits in CAD management fully.

In a similar by Blanco-Colio et al. [21], elevated plasma levels of monocyte chemoattractant protein-1 (MCP-1) were significantly associated with recurrent cardiovascular events, particularly acute coronary syndrome and ischemic stroke, in patients with persistent inflammation indicated by elevated C-reactive protein. This finding underscores MCP-1’s role as a critical marker of cardiovascular risk, especially in inflammatory states. Similarly, our own research identified elevated MCP-1 and NT-proBNP as biomarkers indicating increased inflammation and cardiac dysfunction in elderly coronary heart disease (CHD) patients. Importantly, our intervention, which combined Traditional Chinese Medicine (Baoyuan and Taohong Siwu decoctions) with conventional Western therapy, successfully lowered MCP-1 and NT-proBNP levels, improved lipid profiles, and relieved clinical symptoms. This suggests that therapeutic targeting of inflammatory markers like MCP-1 through integrative approaches could potentially reduce recurrent cardiovascular events in patients with persistent inflammation, complementing and extending Blanco-Colio et al.’s findings by highlighting the clinical utility of integrated therapies.

Similarly, NT-proBNP levels significantly decreased in patients undergoing combined therapy compared to the control group, reinforcing the cardiac-protective effect of integrating BYD-THSWD with Western medication. NT-proBNP, a well-established biomarker for cardiac dysfunction, correlates strongly with myocardial stress and ventricular overload conditions. The greater reduction in NT-proBNP levels observed in the RG group highlights an improvement in cardiac function, possibly due to the multi-targeted cardiovascular protective effects of BYD-THSWD. Specifically, BYD is traditionally recognised for invigorating qi and enhancing myocardial energy metabolism, while THSWD promotes blood circulation, reduces blood stasis, and has cardioprotective and endothelial-reparative actions. Together, these herbal decoctions may mitigate myocardial remodelling and reduce ventricular strain, thus lowering NT-proBNP levels.

Furthermore, this study demonstrated notable improvements in lipid metabolism parameters, including triglycerides (TG), total cholesterol (TC), low-density lipoprotein cholesterol (LDL-C), and high-density lipoprotein cholesterol (HDL-C) in patients receiving the integrated nutritional therapy. Dyslipidemia is a primary risk factor in CHD progression, and effective lipid control remains essential in therapeutic management strategies. The significant reductions in lipid profiles observed in patients treated with BYD-THSWD, beyond those receiving conventional therapy alone, suggest additional metabolic regulatory effects conferred by these decoctions. The lipid-lowering effects of BYD-THSWD may be attributed to components such as Ligusticum wallichii and Safflower, which have documented lipid-regulating and anti-atherosclerotic activities. These herbal components might inhibit lipid absorption, enhance lipid metabolism, or improve endothelial function, contributing to improved lipid profiles observed in this research.

Clinically, patients in the RG demonstrated superior improvements in symptomatology assessed by TCM scores, including reduced chest pain, chest tightness, fatigue, and sweating. These symptomatic improvements, coupled with biochemical evidence, further substantiate the holistic therapeutic potential of TCM decoctions. BYD-THSWD not only targets physiological pathways involved in inflammation, lipid metabolism, and cardiac function but also addresses patient-specific syndromes consistent with Qi deficiency and blood stasis in traditional diagnostic frameworks. This individualised approach enhances patient adherence and may offer broader quality-of-life improvements beyond those achieved by Western medication alone.

However, several limitations must be acknowledged. First, the sample size was relatively small, limiting generalizability and necessitating further studies with larger, more diverse populations. Second, the follow-up duration was limited to three months, precluding the assessment of long-term sustainability and its impact on cardiovascular outcomes, such as myocardial infarction incidence or cardiovascular mortality. Long-term follow-up studies are needed to clarify whether sustained reductions in MCP-1, NT-proBNP, and lipid levels translate into meaningful clinical benefits. Additionally, specific molecular mechanisms underlying the beneficial effects of BYD-THSWD were not elucidated in this investigation and warrant further exploration.

## Conclusion

In conclusion, combining Baoyuan and Taohong Siwu decoctions with Western medicine effectively reduces inflammatory and cardiac biomarkers, improves lipid metabolism, and relieves symptoms in elderly CHD patients. This integrated approach offers a promising complementary therapy for managing coronary heart disease. Further studies are needed to explore its long-term benefits.

## Dodatak

### Acknowledgements

The authors would like to express their gratitude to the medical and nursing staff at Shanghai Eighth People’s Hospital, Huajing Community Health Service Centre, and Central Hospital of Xuhui District for their valuable assistance and support during the study. Special thanks also go to all participants who consented to take part in this research.

### Funding

This research did not receive specific funding from any agencies in the public, commercial, or not-for-profit sectors.

### Ethics statement

The study protocol was approved by the Ethics Committee of Shanghai Eighth People’s Hospital, and all participants provided informed written consent prior to enrollment. The ethical guidelines outlined in the Declaration of Helsinki were strictly followed.

### Authors’ contributions

Z.W. conceived the study, supervised research activities, and provided overall direction. Y.W., W.J., and H.Z. performed data collection and conducted patient assessments and laboratory analyses. W.J. and Y.W. conducted statistical analyses and prepared the initial manuscript draft. Z.W. critically reviewed and revised the manuscript. All authors have read, revised, and approved the final manuscript.

### Data availability

All relevant data supporting the findings of this study are available upon request from the corresponding author.

### Conflict of interest statement

All the authors declare that they have no conflict of interest in this work.

## References

[1] di Cesare M, Perel P, Taylor S, Kabudula C, Bixby H, Gaziano T A, McGhie D V, Mwangi J, Pervan B, Narula J, Pineiro D, Pinto F J (2024). The Heart of the World. Glob Heart.

[2] Gaziano T A, Bitton A, Anand S, Abrahams-Gessel S, Murphy A (2010). Growing Epidemic of Coronary Heart Disease in Low- and Middle-Income Countries. Curr Probl Cardiol.

[3] Sanchis-Gomar F, Perez-Quilis C, Leischik R, Lucia A (2016). Epidemiology of coronary heart disease and acute coronary syndrome. Ann Transl Med.

[4] Jebari-Benslaiman S, Galicia-García U, Larrea-Sebal A, Olaetxea J R, Alloza I, Vandenbroeck K, Benito-Vicente A, Martín C (2022). Pathophysiology of Atherosclerosis. Int J Mol Sci.

[5] Díez-Villanueva P, Jiménez-Méndez C, Bonanad C, García-Blas S, Pérez-Rivera Á, Allo G, García-Pardo H, Formiga F, Camafort M, Martínez-Sellés M, Ariza-Solé A, Ayesta A (2022). Risk Factors and Cardiovascular Disease in the Elderly. Rev Cardiovasc Med.

[6] Deshmane S L, Kremlev S, Amini S, Sawaya B E (2009). Monocyte Chemoattractant Protein-1 (MCP-1): An Overview. J Interferon Cytokine Res.

[7] Makarewicz-Wujec M, Henzel J, Kępka C, Kruk M, Wardziak Ł, Trochimiuk P, Parzonko A, Dzielińska Z, Demkow M, Kozłowska-Wojciechowska M (2021). Usefulness of MCP-1 Chemokine in the Monitoring of Patients with Coronary Artery Disease Subjected to Intensive Dietary Intervention: A Pilot Study. Nutrients.

[8] Gonzalez-Quesada C, Frangogiannis N G (2009). Monocyte chemoattractant protein-1/CCL2 as a biomarker in acute coronary syndromes. Curr Atheroscler Rep.

[9] Cao Z, Jia Y, Zhu B (2019). BNP and NT-proBNP as Diagnostic Biomarkers for Cardiac Dysfunction in Both Clinical and Forensic Medicine. Int J Mol Sci.

[10] Sarzani R, Allevi M, di Pentima C, Schiavi P, Spannella F, Giulietti F (2022). Role of Cardiac Natriuretic Peptides in Heart Structure and Function. Int J Mol Sci.

[11] Mouzarou A, Hadjigeorgiou N, Melanarkiti D, Plakomyti T E (2025). The Role of NT-proBNP Levels in the Diagnosis of Hypertensive Heart Disease. Diagnostics (Basel).

[12] Weber M, Hamm C (2005). Role of B-type natriuretic peptide (BNP) and NT-proBNP in clinical routine. Heart.

[13] Sigamani A, Gupta R (2022). Revisiting secondary prevention in coronary heart disease. Indian Heart J.

[14] Zodda D, Giammona R, Schifilliti S (2018). Treatment Strategy for Dyslipidemia in Cardiovascular Disease Prevention: Focus on Old and New Drugs. Pharmacy (Basel).

[15] Dai J, Qiu L, Lu Y, Li M (2024). Recent advances of traditional Chinese medicine against cardiovascular disease: overview and potential mechanisms. Front Endocrinol (Lausanne).

[16] Meng H, Du Z, Lu W, Wang Q, Sun X, Jiang Y, Wang Y, Li C, Tu P (2021). Baoyuan decoction (BYD) attenuates cardiac hypertrophy through ANKRD1-ERK/GATA4 pathway in heart failure after acute myocardial infarction. Phytomedicine.

[17] Chen R, Song C, Qiu J, Su Q, Wang X, Deng G, Cheng K, Chen X, Xiang W, Liu T, Chen X, Wu J (2023). Exploring the potential mechanism of Taohong Siwu decoction in the treatment of avascular necrosis of the femoral head based on network pharmacology and molecular docking. Medicine (Baltimore).

[18] Balion C, Santaguida P L, Hill S, Worster A, McQueen M, Oremus M, et al. (2006). Testing for BNP and NT-proBNP in the diagnosis and prognosis of heart failure. Evid Rep Technol Assess (Full Rep).

[19] Chen Z, Li C, Yu J (2023). Monocyte chemoattractant protein-1 as a potential marker for patients with sepsis: A systematic review and meta-analysis. Front Med (Lausanne).

[20] Martín-Reyes R, Franco-Peláez J A, Lorenzo Ó, González-Casaus M L, Pello A M, Aceña Á, Carda R, Martín-Ventura J L, Blanco-Colio L, Martín-Mariscal M L, Martínez-Milla J (2016). Plasma Levels of Monocyte Chemoattractant Protein-1, n-Terminal Fragment of Brain Natriuretic Peptide and Calcidiol Are Independently Associated with the Complexity of Coronary Artery Disease. PLoS One.

[21] Blanco-Colio L M, Méndez-Barbero N, Pello Lázaro A M, Aceña Á, Tarín N, Cristóbal C, Martínez-Milla J, González-Lorenzo Ó, Martín-Ventura J L, Huelmos A, Gutiérrez-Landaluce C, López-Castillo M (2021). MCP-1 Predicts Recurrent Cardiovascular Events in Patients with Persistent Inflammation. J Clin Med.

